# Self Representations and Music Performance Anxiety: A Study With Professional and Amateur Musicians

**DOI:** 10.5964/ejop.v14i4.1554

**Published:** 2018-11-30

**Authors:** Claudia Castiglione, Alberto Rampullo, Silvia Cardullo

**Affiliations:** a Department of Clinical and Experimental Medicine, University of Messina, Messina, Italy; b Department of Political and Social Sciences, University of Catania, Catania, Italy; Department of Psychology, Webster University Geneva, Geneva, Switzerland; Maynooth University, Maynooth, Ireland

**Keywords:** self-representations, musical performance, performance anxiety, proximity, semantic space

## Abstract

Individual, social and situational factors might play an important role on the experience of anxiety during musical performances. The present research focused on the relationship between self-representations, including musical self, and performance anxiety among a sample of Italian professional and amateur musicians (N = 100; age, M = 23.40, 50% females). We predicted that higher self-discrepancies (actual vs. future self) would be associated with higher performance anxiety in a musical setting (vs. a non musical one), via musical self, and only in professional musicians. The results confirmed our hypothesis. Higher discrepancies between actual and future self-representations were positively associated with higher performance anxiety levels via the musical self only in participants who play instruments at a professional level. Furthermore, musical self influenced performance anxiety levels in a music related setting (i.e., a concert) but not in a non musical one (i.e., an exam).

Musical performance requires high levels of skill in several areas. Musicians need years of intensive training to develop the required sensorial, cognitive, and behavioural skills (Conrad & Roth, 2007; Kraus & Chandrasekaran, 2010; Standley, 2008). Furthermore, musicians place a great importance on the level and the quality of their performances because they recognize them as a fundamental element of their personal identity (Langendörfer, Hodapp, Kreutz, & Bongard, 2006). Indeed the quality of their performances affects their self-esteem and their career, and, in this context, their general tendency to perfectionism and meticulousness might be considered useful and desirable in the pursuit of their goals (Kirchner, 2003; Langendörfer et al., 2006). Despite the common belief that years of practice could protect musicians from anxiety before or during musical performance, research showed that practice and experience cannot guarantee that musical performances will be free of feelings of anxiety and stress (Nagel, 1990; Taborsky, 2007).

Many world-renowned musicians (e.g., Frederic Chopin, Sergei Rachmaninoff and Barbara Streisand) have experienced music performance anxiety with negative physical and psychological consequences (Brodsky, 1996; Kenny, 2011). It could negatively affect not only the quality of the artist’s exhibition though an increase of anxiety levels before, or during, a performance in front of an audience, but it could have also negative effects on the self-esteem of the artist (Kenny, 2011; Sinden, 1999).

## Performance Anxiety in Musicians

When an individual faces a threat, the human body reacts through a series of physical changes useful to facilitate fight, flight or freeze response behaviours (Barlow, 2002; Maack, Buchanan, & Young, 2015; Martini, Timmons, & Tallitsch, 2012; Sinden, 1999). Anxiety levels have both positive and negative effects on musical performance. Intermediate levels of performance anxiety play an adaptive role, facilitating the use of some coping strategy with positive effect on musical performance (Kenny, Davis, & Oates, 2004; Lehrer, Goldman, & Strommen, 1990; Steptoe & Fidler, 1987; Wolfe, 1989). However, several studies found that music performance anxiety had negative effects on musicians even if they are professional (James, 1998; Kenny et al., 2004; Van Kemenade, Van Son, & Van Heesch, 1995), and not only in training (Boucher & Ryan, 2011; Fehm & Schmidt, 2006). Indeed anxiety was shown to be positively related with poor musical performance experiences (Aderman et al., 1989; Kubzansky & Stewart, 1999) and, as a consequence of poor performance, with unhealthy coping strategies (Park, 2010) with the possibility to end prematurely their musical careers (Ryan & Andrews, 2009). Even so, more experienced students might perform better than less experienced peers under anxiety conditions (Hamann, 1982; Osborne, Kenny, & Holsomback, 2005).

Research on musical performance anxiety has focused on several individual, social and situational factors like motivation, personality traits, emotions, type of instrument, performance setting, presence of an audience or type of performance (Kenny, 2011). Motivations behind musicians’ career path seem to play an important role. Individuals showed higher level of performance anxiety when their motivation was external, like desire to satisfy parents’ wishes (Seamands, 1999). Other individual factors might also play an important role. Perfectionism was shown to be associated with higher levels of anxiety in musicians (Kenny et al., 2004; Mor, Day, Flett, & Hewitt, 1995), especially socially prescribed perfectionism (Langendörfer et al., 2006). Actually, the social aspect of performance seems to be a central aspect in how musicians cope with their anxiety levels. Cox and Kenardy (1993) found that anxiety increased when musicians performed solo on stage compared to practice and group sessions. Indeed the mere presence of other individuals may alter their performance if we consider Triplett’s research (1898) showing that cyclists register better race times when racing with others. Subsequently Zajonc (1968), using the theory of Hull-Spence (1956), extended Triplett’s (1898) results about social influences on performance. According to Zajonc (1968), the mere presence of a public creates a state of excitement resulting in a state of readiness. Thus, social influence can have facilitating or inhibitory effects on performance. The presence of co-actors or a public can improve the speed and accuracy of performance in simple or well-known tasks (social facilitation) and it can compromise performance in complex or poorly learned tasks (social inhibition; Bond & Titus, 1983).

According Zajonc (1965) social facilitation occurs because the presence of others increases the individuals’ physiological activation, which makes some behaviour easier to perform. Physiological activation facilitates performance based on behaviours that are very accessible, simple, well learned or practiced. In opposition, physiological activation can inhibit instead performances based on complex or new behaviours. Social inhibition delineates all the conscious or unconscious avoidance behaviours of a specific situation or of a social interaction when individuals perceive the chance that other people might express feelings of disapproval (Denollet, 2013). Thus, social inhibition has proven to be related to social concerns for evaluations, anxiety about social interactions, social avoidance, and withdrawal (Denollet, 2013). Social inhibition also has an impact on musicians. For example, Brotons (1994) explored the effects of the performance setting (jury and nonjury situation) on music performance anxiety among a sample of music students, covering a wide range of music disciplines. Music performance anxiety was measured through several physiological, behavioural and self-reported measures and participants in a jury setting showed higher heart rates and anxiety levels as compared to a nonjury setting. Similarly, LeBlanc, Jin, Obert, and Siivola (1997) found that the presence of a group of spectators significantly increased anxiety levels and heart rates. Music performance anxiety have been linked to cognitive processes similar to social phobia and was proven to be higher when an audience was present during a solo performance as compared to group performance, in a private setting, and tape recording session (Osborne & Franklin, 2002).

Musicians tend to react differently to an audience depending on their professional level. Music students experienced high levels of anxiety in front of an audience but more experienced students performed better than less experienced peers under anxiety conditions (Hamann, 1982; Osborne, Kenny, & Holsomback, 2005). Additionally, students who want to become professionals showed higher levels of performance anxiety (Robson & Kenny, 2017). A recent study (Barbar, de Souza Crippa, & de Lima Osório, 2014) among Brazilian musicians showed that professional and amateur musicians had similar music performance anxiety, but professional ones showed higher levels of general and social anxiety.

### Self Representations and Anxiety

Representations about oneself shape and affect individuals’ behaviors, cognitions and emotions. These representations might concern beliefs about one’s own actual, ideal, undesired, future, or professional self (Hart et al., 1997; Higgins, Klein, & Strauman, 1985; Markus & Nurius, 1986; Ogilvie, 1987; Strauman & Higgins, 1988). The term future self delineates a set of knowledge about what one might become (Markus & Nurius, 1986). When individuals describe, or think about, themselves, they will use knowledge about characteristics that they believe they actually possess or they will likely acquire in the future. They will use self-knowledge about themselves in the present and projected into the future. This set of knowledge, ‘Possible Selves’ (Markus & Nurius, 1986) refers to the beliefs about how an individual will, or might, become.

Self-representations drive cognitions, emotions and behaviours and facilitate performances by focusing on explicit goals and by strategically implementing plans of actions (Castiglione et al., 2015; Markus & Nurius, 1986; Markus & Ruvolo, 1989). Self-representations are structured in a semantic space (semantic space approach, Hart et al., 1997). Self representations’ descriptors and attributes delimit the position of self representations in the semantic space, the more similarly two self-representations are described or defined, the closer these representations will be located in the semantic space of an individual (Hart et al., 1997; Higgins et al., 1985). Closeness among self-representations might positively affect self-esteem, emotions, and behaviours (Field, Hart, & Horowitz, 1999; Hart & Fegley, 1995; Hart et al., 1997; Heppen & Ogilvie, 2003). Discrepancies and incongruences between self dimensions (Higgins et al., 1985; Markus & Nurius, 1986; Rogers, 1961) showed a negative effect on emotional states like depression, sadness, stress and anxiety (Hart et al., 1997; Heppen & Ogilvie, 2003; Higgins, 1989).

Performances, goals and careers are also affected by self-concept (Oyserman & Markus, 1990). Self-images affect individuals’ cognitions, emotions (including anxiety) and behaviours (Castiglione, Licciardello, & Rampullo, 2015; Hart, Field, Garfinkle, & Singer, 1997; Heppen & Ogilvie, 2003; Higgins, 1989; Markus & Nurius, 1986; Markus & Ruvolo, 1989) and thus, shape musicians’ performances and careers (Schnare, MacIntyre, & Doucette, 2012; Varvarigou, Creech, & Hallam, 2014). Self-representations were shown to play an important motivational role among musicians, with musical self positively affecting performances, goals and careers in musicians (Oyserman & Markus, 1990; Schnare et al., 2012; Varvarigou et al., 2014). The latter have been related also to the performance setting, with musicians showing higher music performance anxiety in a jury situation (Brotons, 1994) or in a solo performance (Osborne & Franklin, 2002) compared to a non-jury situation or a private setting, especially in professional musicians (Osborne et al., 2005).

Some studies have examined the role of self representations in musicians (Schnare et al., 2012; Varvarigou et al., 2014) focusing on other individual and contextual factors, thus the present study aimed to extend previous literature examining the relationship between self representations and anxiety among a sample of Italian professional and amateur musicians.

### Aims and Hypotheses

**Figure 1:**
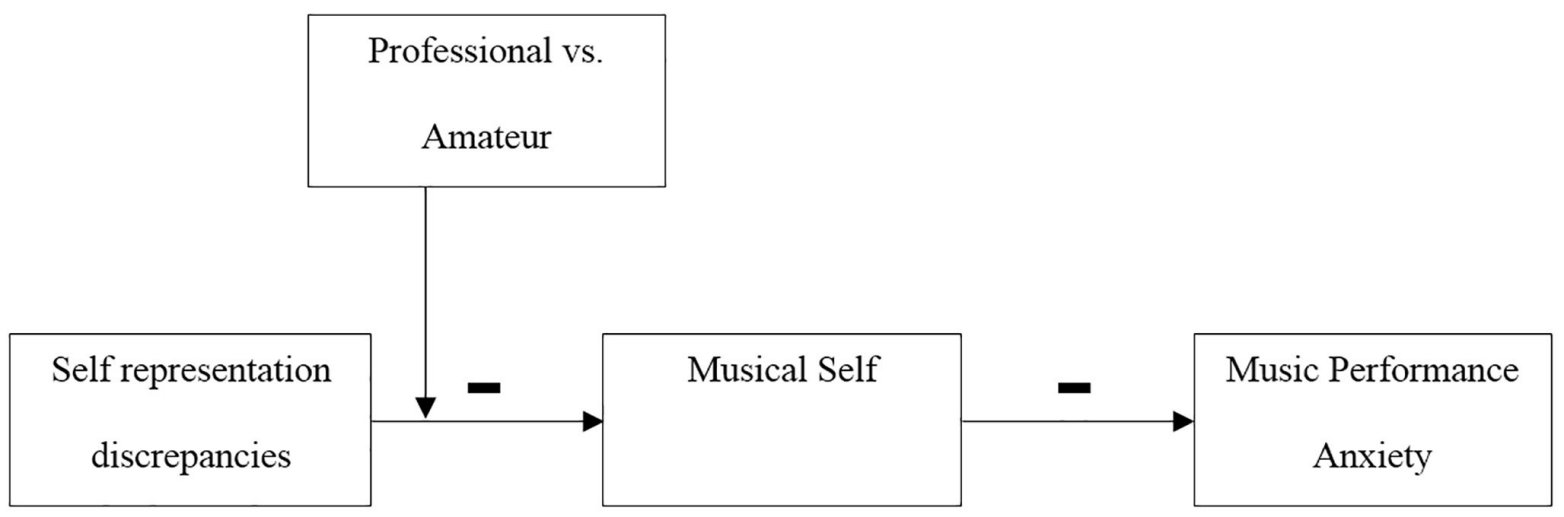
Suggested moderated mediation model. Self-discrepancies (actual vs. future self) associated with music performance anxiety, via the musical self, only in professional musicians.

The present study focused on the relationship between self-representations and anxiety among a sample of Italian professional and amateur musicians. We predicted that self-discrepancies (actual vs. future self) would be negatively associated with musical self, thus higher self-discrepancies would be associated to a less positive representation of the musical self. We expected also that musical self would be negatively associated with music performance anxiety, thus a more positive representation of the musical self would be associated with lower levels of performance anxiety. Finally, we predicted that higher self-discrepancies (actual vs. future self) would be associated with higher performance anxiety in a musical setting as compared to a non-musical one, via the musical self, and only in professional musicians (see Figure 1).

## Method

### Participants

The sample consisted of 100 musicians (*n* = 50 professionals, *n* = 50 males) with an average age of 23.40 (*SD* = 3.80; range 18-30) and an average of 11.26 years of playing an instrument (*SD* = 4.80; range 2-23).

### Measures

#### Performance Anxiety

Performance Anxiety was measured using an Italian Validated version (Pedrabissi & Santinello, 1989) of the State Anxiety Inventory (Spielberger, 1983). It is a self-report questionnaire consisting of 40 items rated on a 6-point Likert scale (1-never to 6-always) about anxiety levels in different social situations. The two social situations we used for the purpose of this research were: a university exam (an oral exam in front of other students; Exam, α = .90) and a concert (α = .88). Both are performance situation in front of an audience, but only the concert situation is a music performance. Higher score refers to higher levels of performance anxiety.

#### Self Representations

Two semantic differentials were used to measure actual and future self (Osgood, Suci, & Tannenbaum, 1957). They were made up of 34 7-point bipolar scales (e.g., strong – weak). This version was already tested in the Sicilian context (see Castiglione, Licciardello, Mauceri, & Rampullo, 2012; De Caroli & Sagone, 2012). It was used to measure the representations of actual self (me as I am) (α = .86), future self (me as I will be) (α = .89), and musical self (the musician) (α = .85). Based on the semantic space approach (Hart et al., 1997), the location of self-representations in individuals’ semantic space is based on the similarity of the self-representations’ descriptors or attributes (Hart et al., 1997; Higgins et al., 1985). Thus, discrepancy was operationalized as Euclidean distance (Hafdahl, Panter, Gramzow, Sedikides, & Insko, 2000; Kirchler, Palmonari, & Pombeni, 1994) between descriptors of the actual and future self and using the Pythagorean Theorem (square root of the summed squared differences between each item/adjective pair). A value of 0 refers identical description of actual and future self, higher values refers to higher discrepancy between them.

#### Background Questions

Background questions were used to get age and gender information of our participants.

## Results

We conducted a series of independent sample t-tests to verify professional level (professional vs. amateur) effects on self-representations discrepancies (actual vs. future self), musical self, and performance anxiety (exam and concert).

Professional level showed a significant effect on self-representations discrepancies (actual vs. future self) *t*(98) = 4.757, *p* < .001 and on concert performance anxiety *t*(98) = 2.222, *p* = .03. Professional participants showed higher self-representations discrepancies and concert performance anxiety compared to amateur participants (see Table 1).

**Table 1:** Means, Standard Deviations and Correlations

Variable	*M*	*SD*	1	2	3	4
Amateur						
1. Actual self vs future self discrepancies	1.15	.82	–			
2. Musical self	5.07	.50	-.07	–		
3. Exam performance anxiety	54.74	10.52	-.48**	-.07	–	
4. Concert performance anxiety	48.24	8.26	.30*	-.47**	.18	–
Professional						
1. Actual self vs future self discrepancies	1.81	.55	–			
2. Musical self	4.90	.74	-.34*	–		
3. Exam performance anxiety	57.95	10.41	.33*	-.12	–	
4. Concert performance anxiety	52.28	9.85	.13	-.50**	.38**	–
Overall						
1. Actual self vs future self discrepancies	1.47	.77	–			
2. Musical self	4.99	.64	-.23*	–		
3. Exam performance anxiety	56.35	10.54	-.44**	-.12	–	
4. Concert performance anxiety	50.26	9.27	.28**	-.50**	.31**	–

**p < .01. *p < .05.

In Amateur participants, we found medium negative correlations between self discrepancies and exam performance anxiety. Furthermore, we found medium negative correlation between musical self and concert performance anxiety. In Professional participants, self discrepancies were negatively related with musical self and positively with exam anxiety. Musical self was also negatively related with concert performance anxiety (see Table 1).

Zero order correlations, means and standard deviations for self-representations discrepancies (actual vs. future self), musical self, and performance anxiety (exam and concert) are shown in Table 1.

To test our hypothesis we followed Preacher, Rucker, and Hayes (2007) approach to moderated mediation analysis. Initially, we tested if the path from the predictor (i.e., self-discrepancies) to the mediator (i.e., musical self) was affected or not by the proposed moderator professional level (professional vs. amateur). Subsequently, we tested the path between the mediator and the outcome measures (performance anxiety in musical and non-musical setting). Finally, the moderated mediation model was test through PROCESS macro, Model #7 (Hayes, 2013).

Firstly, we tested through a multiple hierarchical regression analyses (Baron & Kenny, 1986) the effects of the predictor (self discrepancies) on the proposed mediator (musical self) and the interaction between the predictor and the professional level (professional vs. amateur). Variables were centred to avoid multicollinearity (Cohen, Cohen, West, & Aiken, 2003). To test the interaction between the predictor (self discrepancies) and professional level, one interaction term was created. If the interaction term significantly contributed to the regression model, we proceeded to investigate conditional effect of self-discrepancies on musical self at moderator values (professional vs. amateur).

**Figure 2:**
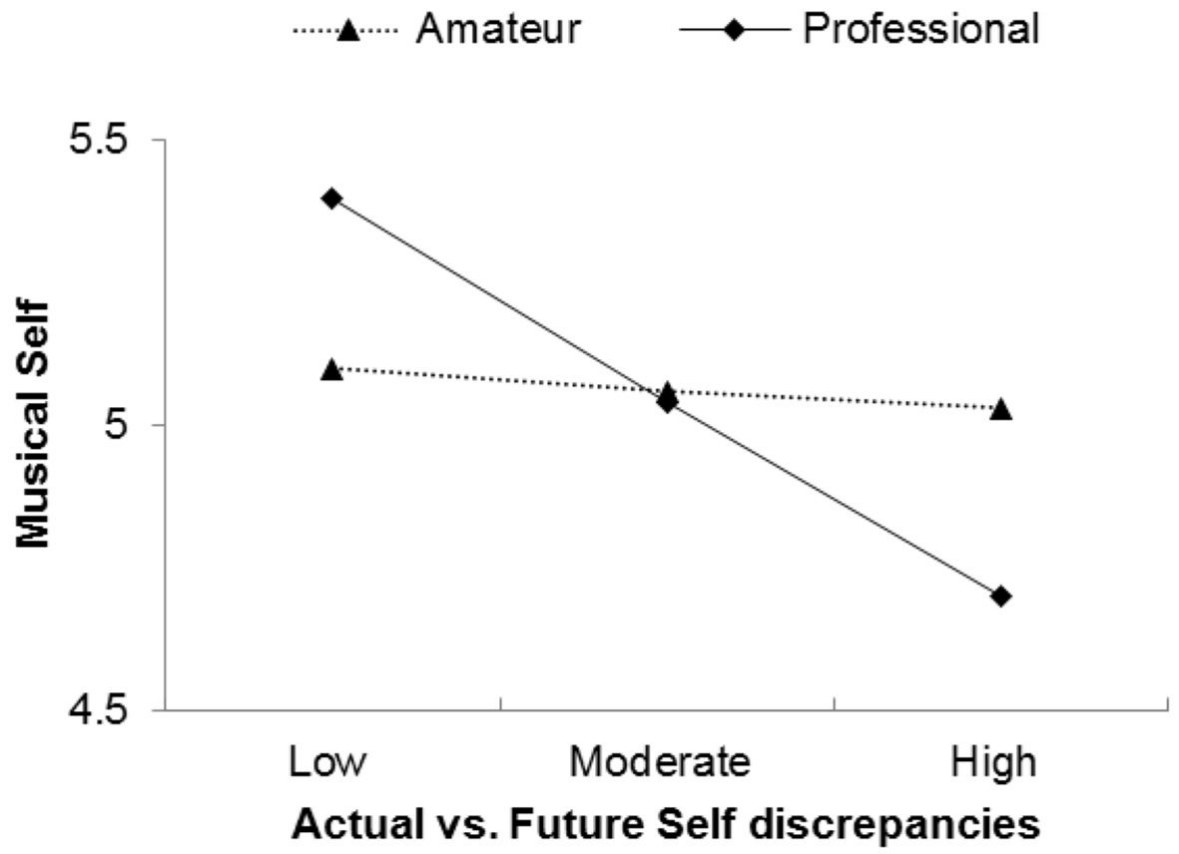
Musical self levels as a function of actual vs. future self-discrepancies and professional level.

We tested the effects of self-discrepancies and the professional level on musical self. Self discrepancies showed a significant and negative main effect, *b* = -.25, *t*(98) = -3.066, *p* = .003 on musical self, *R^2^*_adj_ = .10, *F*(3, 96) = 4.67, *p* = .004. The addition of the interaction term significantly contributed to the regression model, *R^2^_change_* = .04, *F_change_* (1, 96) = 6.39, *p* = .01. The interaction between self discrepancies and the professional level was significant, *t*(96) = -2.528, *p* = .01, suggesting that the effect of self discrepancies on musical self depends on professional level. Simple slopes analysis showed that self-discrepancies did not significantly affect musical self for amateur musicians. On the other hand, high discrepancies between actual and future self had a significant negative effect on musical self-representation for professional musicians, *b* = -.45, *SE* = .14, *t*(96) = -3.189, *p* = .002 (see Figure 2).

After testing the path between the predictor and the proposed mediator, we tested the paths between musical self representation and performance anxiety (exam and concert) through a series of regression analyses with musical self representation and professional level entered simultaneously in the model. Only musical self-representation had significant influence on concert performance anxiety: more positive musical self-representation was significantly associated with lower concert performance anxiety, *b* = -6.94, *t* = -5.433, *p* < .001, *R^2^*_adj_ = .27, *F*(2, 97) = 17.944, *p* < .001. As hypothesized, musical self-representation and professional level were not significantly associated with performance anxiety in a non-musical setting (exam).

Finally, to test if the indirect effects of self-discrepancies on performance anxiety through musical self (mediator) depend on professional level or not, we used the Model 7 of the PROCESS macro (Hayes, 2013). We tested the moderated mediation model with concert performance anxiety as outcome variable using bootstrapping with 5,000 resamples. The index of moderated mediation was significant (Hayes, 2013), index = 2.73, *SE* = 1.23, 95% CI [.80, 5.65], indicating that professional level moderated the mediation effect of musical self on the relationship between self-discrepancies and concert performance anxiety. Specifically, the indirect effect of self-discrepancies on concert performance anxiety via musical self was significant only for professional musicians, ab = 3.02, *SE* = 1.21, 95% CI [1.09, 5.99].

## Discussion

Studies on musicians’ performance anxiety have explored different individual, social and situational variables like motivation, personality, emotions, or performance setting (Kenny et al., 2004; LeBlanc et al., 1997; Mor et al., 1995; Seamands, 1999). Performance setting (like jury or non-jury situation) might affect how the musician experiences the performance and the levels of anxiety that they feel (Brotons, 1994; Osborne & Franklin, 2002; Osborne et al., 2005). However, musicians might react differently based on their professional level, with professionals experiencing higher levels of anxiety (Osborne et al., 2005) even if they have been shown to cope better under anxiety conditions (Hamann, 1982). Finally, self-representation might affect performance (Schnare et al., 2012; Varvarigou et al., 2014) and anxiety levels (Hart et al., 1997; Heppen & Ogilvie, 2003; Higgins, 1989) also in musicians.

The present research focused on the relationship between self-representations and anxiety among a sample of Italian professional and amateur musicians. More specifically, we predicted that higher self-discrepancies (actual vs. future self) would be associated with higher performance anxiety in a musical setting as compared to a non-musical one, via the musical self, and only in professional musicians.

The results supported our prediction that self-discrepancies (actual vs. future self) were negatively associated with the representation of musical self, thus higher discrepancy between actual and future self was associated with less positive representation of musical self. These results seems support the motivational role of possible selves (Markus & Nurius, 1986), especially if we consider that the negative association between self discrepancies and musical self emerged only for professional musicians. Varvarigou et al. (2014) already described how supporting possible selves through positive role models elicited detailed descriptions of students’ musical self and a positive motivational effect toward musical activities.

Coherently with previous results and with our predictions, musical self was negatively associated with performance anxiety only in a musical setting (concert). These results support the idea that performance setting could play an important role. Previous research (Brotons, 1994; Osborne & Franklin, 2002; Osborne et al., 2005) already showed how musicians reported different level of anxiety based on the performance setting (e.g. jury – non-jury situation). Finally, in line with literature on self dimensions (Castiglione et al., 2015; Markus & Nurius, 1986; Markus & Ruvolo, 1989), and with literature on differences between professional and amateur musicians (Hamann, 1982; Osborne et al., 2005), higher discrepancies between self-representations were associated with higher performance anxiety levels via the musical self only in participants who play instruments at a professional level. Furthermore, coherently with literature on effects of the settings on music performance anxiety (Osborne & Franklin, 2002), musical self was associated with performance anxiety only in a music related setting. Results suggests that musical self might play an important role as a mechanism through which musicians could more easily manage performance anxiety in musical performance setting.

### Limits of the Study

A major concern of the present study was that music performance anxiety was measured with only a self-reported measure. Future studies should explore the effect of self dimensions on anxiety levels through the use of physiological and behavioural measures (Brotons, 1994; LeBlanc et al., 1997). Furthermore, data showed a positive correlation between exam and concert performance anxiety, thus indicating that anxious people might be more anxious no matter the context. Nevertheless musical self representation and professional level were not significantly associated with performance anxiety in a non-musical setting (exam), indicating that the setting might play, at least in part, an important role.

Another limit is related to the use of a correlational design. Previous studies have addressed already the causal path linking self-images discrepancies to anxiety (Heppen & Ogilvie, 2003) employing different research designs (for example, a longitudinal design). Future studies should implement also other research designs (for example, an experimental design). Future studies could consider also other factors central for professional development and representation of self-dimensions like creativity (De Caroli, Licciardello, Sagone, & Castiglione, 2010; Gladding, 2008) or self-efficacy (Brouwers & Tomic, 2000; Skaalvik & Skaalvik, 2007).

### Conclusion

The results of the present study showed for the first time that self-representations discrepancies were associated with performance anxiety in a musical setting (vs. a non musical one), via musical self, only in professional musicians, thus extending previous literature on the relationship between possible selves (Markus & Nurius, 1986) and music performance anxiety. The results also seem fundamental for the literature on educational intervention to promote and sustain a musical career with youth scholars (Varvarigou et al., 2014). Intervetions focused only on contextual factors should include individual variables through the employment of strategies aimed at reducing self-images discrepancies and sustaining a positive musical self representation. Professional psychological support might be directed on both improvement of own self-image and supporting a positive representations of musicians as a professional group.
